# Antiferroelectric thin films embedded with ferroelectric switching loop for giant negative electrocaloric effect

**DOI:** 10.1126/sciadv.aed5447

**Published:** 2026-07-01

**Authors:** Peipei Su, Jianchu Chen, Junning Li, Jinbin Wang, Zhenzhong Yang, Yongshuai Ge, Ke Qu, Xiangli Zhong, Gaokuo Zhong

**Affiliations:** ^1^ Key Laboratory of Low Dimensional Materials and Application Technology of Ministry of Education, School of Materials Science and Engineering, Xiangtan University, Xiangtan 411105, Hunan, China.; ^2^ Changsha Semiconductor Technology and Application Innovation Research Institute, College of Semiconductors (College of Integrated Circuits), Hunan University, Changsha 410082, China.; ^3^ Shenzhen Institute of Advanced Technology, Chinese Academy of Sciences, Shenzhen 518055, Guangdong, China.; ^4^ Key Laboratory of Polar Materials and Devices, Ministry of Education, Department of Electronics, East China Normal University, Shanghai 200241, China.

## Abstract

The ferroics combine the single-hysteresis loop of ferroelectrics with the double-hysteresis loop of antiferroelectrics to form multiple hysteresis loops, which could substantially advance energy storage, electrocaloric cooling, and nonvolatile multistate memory technologies. However, the intentional stabilization of intermediate states that bridge the nonvolatility of ferroelectrics and the field-induced phase transition behavior of antiferroelectrics remains a fundamental challenge. Here, we propose a strategy for preparing lead zirconate (PbZrO_3_) thin film at low temperature, introducing a stable ferrielectric phase within the antiferroelectric to achieve triple-hysteresis loop under large electric fields. Microstructural features reveal that this behavior is attributable to the presence of Pb_Zr_ antisite defects acting as seeds for polar order, which induce the distinctive triple (↑↑↓) dipole modulation period configuration. To demonstrate the application potential, we evaluated the electrocaloric effect of triple-hysteresis PbZrO_3_ thin film based on Maxwell’s relations, the predicted temperature change Δ*T* can reach −23.76 kelvins, which is ~600% enhancement compared to double-hysteresis PbZrO_3_ antiferroelectric thin films. These findings establish a design paradigm for embedding stable ferroelectric switching within antiferroelectrics, which may unlock opportunities for developing high-density energy storage, nonvolatile multistate memory, and highly efficient switching devices.

## Teaser

The antiferroelectric thin film with triple-hysteresis loop achieves giant negative electrocaloric effect.

## INTRODUCTION

**Fig. 1.**
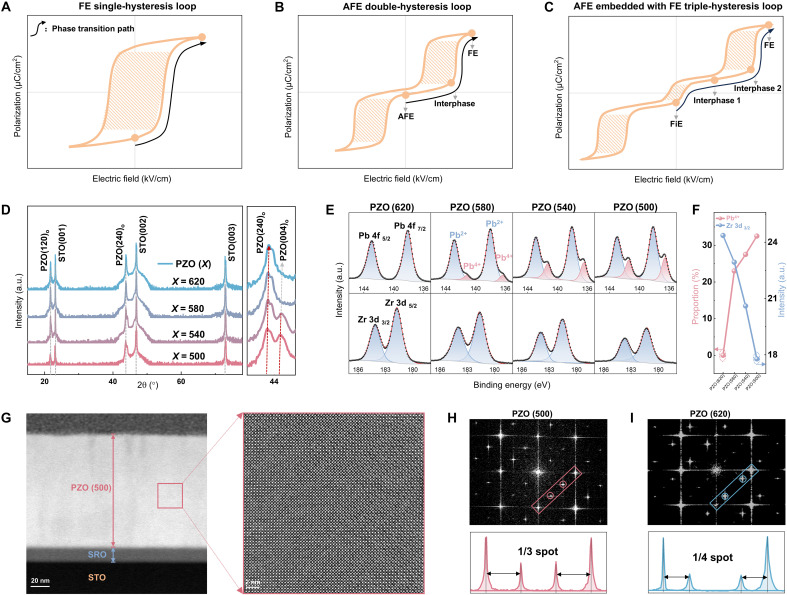
Structural characterization of PZO (620), PZO (580), PZO (540), and PZO (500) thin films. (**A** to **C**) Schematic diagrams of single-, double-, and triple-hysteresis loops, respectively. (**D**) X-ray diffraction (XRD) patterns of PZO (620), PZO (580), PZO (540), and PZO (500) thin films. (**E**) X-ray photoelectron spectroscopy (XPS) spectra of the Pb 4f and Zr 3d in PZO (620), PZO (580), PZO (540), and PZO (500) thin films. (**F**) The statistics of Pb^4+^ proportion and Zr 3d 3/2 peak intensity. (**G**) Large-scale dark-field TEM image of PZO (500) thin film and atomic-resolution STEM image from the region in the pink box marked. [The left panel of (G) is identical to the left panel of fig. S1A.] (**H** and **I**) The corresponding fast Fourier transform (FFT) patterns and intensity profile of the selected rectangular regions for PZO (500) and PZO (620) thin films, respectively. a.u., arbitrary units.

Ferroic materials are characterized by fast dipole switching and electrically tunable domain structures (*1*–*3*), offering broad application prospects in energy storage capacitors, nonvolatile memories, and electrocaloric cooling devices (*4*–*6*). Ferroelectrics (FEs) display a single-hysteresis loop arising from their switchable nonvolatile spontaneous polarization (Fig. 1A), which confers advantages including rapid switching speed, long-term data retention capability, low power consumption, excellent fatigue endurance, and radiation resistance (*7*–*9*). By contrast, antiferroelectrics (AFEs) exhibit the characteristic of double-hysteresis loop due to the field-induced reversible phase transition between an antipolar AFE ground state and a polar FE phase (Fig. 1B) (*10*, *11*). The near-zero remanent polarization of ideal AFEs results in volatile behavior (*12*, *13*), while the field-induced phase transition feature effectively enhances energy storage density, enables multistate memory functionality, and improves electrocaloric cooling efficiency (*14*–*16*). Therefore, combining the nonvolatility of FEs with the field-induced phase transition of AFEs offers a promising strategy for designing ferroics with multiple hysteresis loops, which may yield richer physical properties and broader application prospects (Fig. 1C) (*17*–*19*). Such multiple hysteresis behavior enhances the phase transition electric field and introduces stable intermediate states, thereby improving energy storage performance and permitting nonvolatile multistate memory (*20*–*24*). Multilevel phase transitions produce larger changes in dipolar entropy and lattice structure while raising the electric field required for the AFE-to-FE phase transition (*25*, *26*). Both factors contribute to the enhancement of the negative electrocaloric effect (ECE) in accordance with Maxwell’s relations (*27*–*29*).

In desirable ferroic materials exhibiting multilevel phase transitions, it is critical to delicately design the coexistence between competing FE and AFE phases while precisely regulating the phase transition dynamics (*30*, *31*). However, the intentional stabilization of intermediate states that bridge the nonvolatility of FEs and the field-induced phase transition behavior of AFEs remains a fundamental challenge. Recently, researchers have used microscopic techniques to detect the ferrielectric phases in AFEs with nonzero amplitude response and unique dipole arrangement (*32*, *33*). In addition, field-induced phase transition pathways have been revealed through atomic-level detection and theoretical simulations (*32*–*34*). Subsequent studies have confirmed that the emergence and stability of ferrielectric phases can be systematically tuned by structural defects (*35*, *36*), crystal orientation (*37*, *38*), size effect (*39*, *40*), epitaxial strain (*41*, *42*), and chemical composition (*43*, *44*). These results reveal that complex phase transition dynamics are governed by the interplay among various structural parameters and competing phase formation energies. Representative advances include theory-guided design of NaNbO_3_ thin films with competing AFE orthorhombic and FE rhombohedral phases (*45*), composition-driven three-step domain evolution in Hf_1−*x*_Zr*_x_*O_2_ thin films (*46*), and defect-engineered multiple field-induced transitions in high energy storage (Pb,La)(Zr,Sn,Ti)O_3_ system (*47*). Despite these insights, most current research still primarily focused on elucidating the structural characteristics of ferrielectric phase from the microscopic perspective. There remains a notable scarcity of reports exploring the functional implications following the intentional incorporation of the ferrielectric phase into AFE matrices, leaving a critical gap between microscopic understanding and macroscopic application.

In this work, we designed the low-temperature deposition strategy to obtain the triple-hysteresis PbZrO_3_ thin film with a stable FE switching loop, which introduces the multilevel phase transitions and improves the AFE-to-FE phase transition electric field. Diffraction and photoelectron spectroscopy combined with electron microscopy characterizations reveal that this behavior is attributable to the presence of Pb_Zr_ antisite defects acting as seeds for polar order, which induce the distinctive triple (↑↑↓) dipole modulation period configuration. To demonstrate the functional potential of this behavior, we evaluated the ECE of triple-hysteresis PbZrO_3_ thin film based on Maxwell’s relations. The predicted temperature change Δ*T* can reach −23.76 K, ~600% improvement compared to double-hysteresis PbZrO_3_ AFE thin films, which indicates that introducing the multilevel phase transitions and improving the phase transition electric field can enhance the negative ECE. Furthermore, the triple-hysteresis thin film exhibits low hysteresis loss over a broad temperature range, holding promise for achieving highly efficient electrocaloric cycles and superior energy storage performance (*48*, *49*). These results not only establish a design paradigm for introducing stable FE switching in AFEs but also pave the way for applications in high-density energy storage, nonvolatile multistate memory, and highly efficient switching devices.

## RESULTS

### Structural characterizations of PbZrO_3_ thin films

To obtain ferroics with multilevel phase transitions, PbZrO_3_ thin films were grown by pulsed-laser deposition (PLD) under four different deposition temperatures (620°, 580°, 540°, and 500°C). Figure 1D shows the crystalline structure of the PbZrO_3_/SrRuO_3_/SrTiO_3_(001) heterostructures was characterized by x-ray diffraction (XRD). Bulk PbZrO_3_ exhibits an orthorhombic unit cell (lattice parameters *a*_o_≈$\sqrt{2}$*a*_pc_ ≈ 5.884 Å, *b*_o_ ≈ 2$\sqrt{2}$*a*_pc_ ≈ 11.768 Å, and *c*_o_ ≈ 2c_pc_ ≈ 8.220 Å, where “o” and “pc” denote orthorhombic and pseudocubic cells, respectively) (*50*). The PbZrO_3_ thin films deposited under high temperature (620° and 580°C) are dominated by the (240)_o_-oriented [i.e., (100)_pc_- or (010)_pc_-oriented], whereas lowering the deposition temperature to 540° and 500°C leads to the coexistence of (240)_o_ and (004)_o_ [i.e., (001)_pc_] orientations (partial enlargement on the right of Fig. 1D). Notably, the PZO(240)_o_ diffraction peak shifts toward lower angles with decreasing deposition temperature, indicating an increase in the spacing of the crystal planes. To elucidate the origin of these structural changes, we probed the electronic states of Pb and Zr by x-ray photoelectron spectroscopy (XPS), as shown in Fig. 1E. In the PZO (620) thin film, the Pb^2+^ doublet (Pb 4f 5/2 at 142.98 eV and Pb 4f 7/2 at 138.08 eV) is visible, corresponding to lead in the PbZrO_3_ lattice. In contrast, in the PZO (580) thin film, besides the Pb^2+^ double peaks, two additional peaks are discernible at 141.18 and 136.18 eV, respectively, which could be attributed to Pb^4+^ (*51*). This speculation derives from previous research indicate that in situ growth conditions can promote partial incorporation of Pb in higher oxidation state on B-site positions (*52*). The proportion of Pb^4+^ gradually increases as the deposition temperature decreases, specifically rising from 23 to 27.5% and then to 32.5% as shown in Fig. 1F. This is attributed to the partial suppression of Pb volatility as deposition temperature decreases, resulting in relatively higher Pb content in PbZrO_3_ thin films. However, the reduced atomic mobility at low temperatures indicates that Pb ions kinetically “freeze” on the nearest Zr sites, rather than migrating to more distant free Pb sites owing to insufficient migration momentum (*53*). Furthermore, first-principles density functional theory calculations reveal that neutral charge state Pb_Zr_ antisite defects are the predominant defects under oxidizing and low-temperature conditions (*54*, *55*). It can be seen from the lower part of Fig. 1E that the peaks of Zr 3d 3/2 and Zr 3d 5/2 are observed around 183.9 and 181.5 eV, respectively, whereas the intensity of both peaks diminishes progressively with decreasing deposition temperature. The reduction of peak intensity is assumed to result from the occupation of Zr sites by Pb ions. Because Pb^2+^ (~1.19 Å) and Pb^4+^ (~0.775 Å) have larger ionic radii than Zr^4+^ (~0.72 Å), the occupation of Zr sites by Pb ions induces local lattice expansion, which explains the mentioned increase of crystal planes spacing.

To further recognize the microstructures of PbZrO_3_ thin films deposited at different temperatures, fig. S1 presents the cross-sectional dark-field transmission electron microscopy (TEM) images of PZO (500) and PZO (620) thin films alongside corresponding elemental energy-dispersive spectroscopy (EDS) analysis. The results demonstrate that both exhibit high epitaxial quality, chemical uniformity, and sharp heterointerfaces. The atomic-resolved high-angle annular dark-field (HAADF)–scanning TEM (STEM) image of the pink-framed region in the PZO layer of PZO (500) thin film (Fig. 1G), displaying high crystalline quality without visible defects. Figure 1 (H and I) corresponds to the fast Fourier transform (FFT) patterns of HAADF-STEM images from selected regions of PZO (500) and PZO (620) thin films respectively, both revealing the additional 1/*x*{011} diffraction spots. It is noteworthy that the additional diffraction spots highlighted by pink circles in Fig. 1H appear further away from the main diffraction spots than the additional diffraction spots indicated by blue circles in Fig. 1I. Figure S2 quantifies the positions and intensity distributions of the main and additional diffraction spots. The additional spots in the PZO (500) thin film occur at ~1/3 (2/3) positions, whereas those in PZO (620) thin film appear at 1/4 (3/4) positions, and the intensity of additional diffraction spots is substantially higher for PZO (500). Consequently, we speculate that the structure of PZO (620) thin film is analogous to a typical periodically symmetric modulation structure, whereas the structure of PZO (500) thin film may be a shorter period and asymmetric modulation structure.

### Atomic-scale characterizations of PZO (620) and PZO (500) thin films

**Fig. 2.**
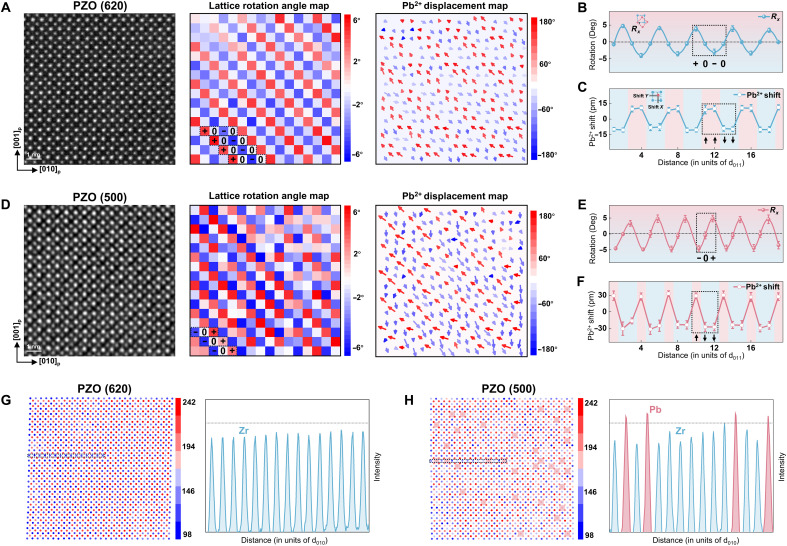
Atomic structure analysis of PZO (620) and PZO (500) thin films. (**A**) Atomic-scale HAADF-STEM image of PZO (620) thin film, corresponding maps of horizontal lattice rotation angle and Pb^2+^ displacement. (**B** and **C**) *R_x_* spacing profiles and shift of Pb cations in PZO (620) thin film. The insets in (B) and (C) define lattice rotation and Pb^2+^ displacements, respectively. (**D**) Atomic-scale HAADF-STEM image of PZO (500) thin film, corresponding maps of horizontal lattice rotation angle and Pb^2+^ displacement. (**E** and **F**) *R_x_* spacing profiles and shift of Pb cations in PZO (500) thin film. (**G**) The Pb and Zr atomic intensity statistics corresponding to atomic-scale HAADF-STEM images in PZO (620) thin film, along with atomic intensity statistics in the black dashed box. (**H**) The Pb and Zr atomic intensity statistics corresponding to atomic-scale HAADF-STEM images in PZO (500) thin film, along with atomic intensity statistics in the black dashed box. Red boxes inside indicate the presence of Pb_Zr_ antisite defect areas.

To probe both dipole configurations and lattice structures, the atomic-resolved HAADF-STEM images were acquired for PZO (620) and PZO (500) thin films. Figure 2A displays the HAADF-STEM image of the PZO (620) thin film together with the maps of horizontal lattice rotation angle and A-site displacement. Quantitative analysis based on two-dimensional Gaussian peak fitting was used to obtain the lattice rotation distribution along the horizontal direction (*R_x_*), assuming that lattice rotation behaviors are indexed by the symbols +, −, and 0. Specifically, the lattice rotation magnitude sequence along the [010]_p_ direction approximates the (+, 0, −, 0) quadruple modulation period. The positions of atomic columns were obtained by Gaussian fitting, and the Pb displacement was calculated by referencing to the average of the surrounding four Zr positions, visualized by arrows (↑, ↓). The Pb^2+^ displacement map intuitively reflects the quadruple modulation configuration with antiparallel arrangement (↑↑↓↓) consistent with the prototype AFE phase. Figure 2B details the average and standard deviation of the lattice rotation angle magnitude. The inset illustrates the definition of lattice rotation, where positive value denotes reverse lattice rotation. The integral exhibits the regular quadruple-periodic distribution, and the modulation magnitude within the black-boxed region is (+3.8°, 0, −3.0°, 0). Figure 2C further extracts the average and standard deviation of the Pb^2+^ displacement amplitude along the [011]_p_ direction from the vector diagram. The overall structure presents the quadruple modulation configuration with antiparallel arrangement (↑↑↓↓), and the displacement amplitude in the black-boxed region is (9.0, 10.0, −8.9, −9.7). The amplitude difference between positive and negative displacement prevents complete offsetting of the dipoles, which may be observed with the nonzero remanent polarization in the hysteresis loop. Figure 2D exhibits the HAADF-STEM image of the PZO (500) thin film, alongside the maps of horizontal lattice rotation angle and A-site displacement. The sequence of lattice rotation magnitude along the [010]_p_ direction can be represented as (−, 0, +) triple modulation period. The Pb^2+^ displacement map follows the triple modulation configuration (↑↓↓), which verifies the previous conjecture derived from diffraction spots. Figure 2E demonstrates that the average magnitude of the lattice rotation angle oscillates around ±4.8°, with slight variations in rotation angle across different regions. The polarization amplitude shown in Fig. 2F oscillates at ~30 pm in the positive direction and around 25 pm in the negative direction. By comparison, the lattice rotation angle magnitude and polarization modulation amplitude of the PZO (500) thin film are both larger than those of the former, consistent with the stronger 1/3 diffraction spots (versus 1/4) observed in the FFT patterns.

We would term the triple dipole modulation period configuration distinct from classical AFE structure as “ferrielectric”, which may be related to specific structural features within the PbZrO_3_ thin film. To gain deeper insight into the ferrielectric phase, we statistically analyzed the atomic intensity of large region atomic images for PZO (620) and PZO (500) thin films in fig. S3. Figure 2 (G and H, left) displays the corresponding atomic intensity maps for both HAADF images. Because the atomic intensity in HAADF images depends on the atomic number (*Z*), a color map is used, with red representing atoms with a higher Z, e.g., Pb atoms at A sites, and with blue representing atoms with a lower *Z*, e.g., Zr atoms at B sites. It should be noted that abnormally high intensities (marked by light red boxes) appear on certain atomic columns at the B sites of the PZO (500) thin film, which are normally occupied by lighter Zr atoms. Quantification of the boxed regions (Fig. 2, G and H, right) indicates the anomalous enhancement of B-site intensity in PZO (500) thin film, suggesting the presence of heavier Pb ions at several Zr-like sites. Note that the valence state of Pb ions occupying the Zr sites can increase to 4+ to maintain charge neutrality in the system without adding compensating charge carriers or point defects. It further confirms that the presence of Pb^4+^ observed in the XPS results is attributed to the several Pb_Zr_ antisite defects in PbZrO_3_ thin film, which gradually increase in proportion as the deposition temperature decreases. Collectively, these observations reveal the mechanism linking Pb_Zr_ antisite defects to the triple modulation configuration (↑↓↓) in low-temperature–deposited PbZrO_3_ thin film.

### Electrical properties of PbZrO_3_ thin films

**Fig. 3.**
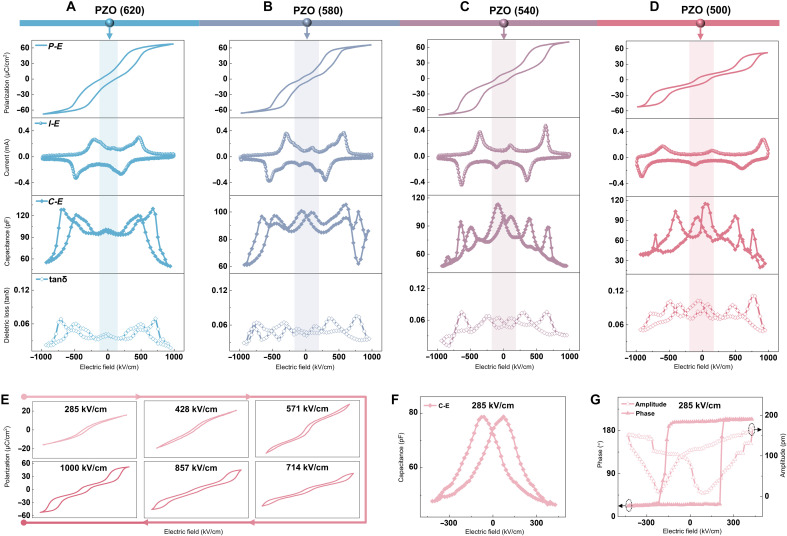
Electrical properties of PZO (620), PZO (580), PZO (540), and PZO (500) thin films. (**A** to **D**) Polarization-electric field (*P-E*) hysteresis, corresponding current-electric field (*I-E*) switching, capacitance-electric field (*C-E*), and dielectric loss (tanδ) loops of the PZO (620), PZO (580), PZO (540), and PZO (500) thin films, respectively. (**E**) Polarization-electric field (*P-E*) hysteresis loops of the PZO (500) thin film evolution with electric fields. (**F**) Capacitance-electric field (*C-E*) loop of the PZO (500) thin film under 285 kV/cm. (**G**) Phase and amplitude hysteresis loops of the PZO (500) thin film under 285 kV/cm.

To evaluate the influence of deposition temperature on the electrical properties of PbZrO_3_ thin films, we measured the polarization-electric field (*P-E*), corresponding current-electric field (*I-E*), capacitance-electric field (*C-E*), and dielectric loss (tanδ) loops. The *P-E* loop of PbZrO_3_ thin film deposited at 620°C (Fig. 3A) displays a typical double-hysteresis loop, while the accompanying *I-E* curve reveals four peaks associated with the AFE-to-FE and FE-to-AFE phase transitions. The nonzero remanent polarization arises from the above dipole displacement amplitudes not completely offsetting in the positive and negative directions. The *C-E* loop shows four peaks indicative of a notable capacitance signal enhancement during AFE and FE transitions, which further corroborates its antiferroelectricity. As shown in Fig. 3B, the *P-E* loop of PbZrO_3_ thin film deposited at 580°C presents a slight fluctuation near zero electric field, with the *I-E* and *C-E* curves also having two weak peaks around zero electric field. This feature becomes more enhanced for PbZrO_3_ thin film deposited at 540°C (Fig. 3C), where fluctuation near zero electric field is more pronounced as seen from the *P-E* loop. The corresponding *I-E* and *C-E* characteristics reveal two peaks at *E*_C_ (about ±100 kV/cm) superimposed on the four typical AFE switching peaks. The *P-E* loop of PbZrO_3_ thin film with deposition temperature reduced to 500°C (Fig. 3D) exhibits a distinct hysteresis within ±120 kV/cm, embedded within the double-hysteresis loop. This hysteresis increases the phase transition electric fields for FE-to-AFE and AFE-to-FE to ±610 and ± 905 kV/cm, respectively. The accompanying *I-E* and *C-E* curves also exhibit six distinct peaks, confirming that the reduced deposition temperature enables additional phase transitions beyond the AFE-to-FE and FE-to-AFE transitions. Notably, the dielectric loss curves match the peaks of the capacitance response for thin films deposited at different temperatures, consistent with the polarization switching behavior. Moreover, the dielectric loss remains of the same order of magnitude across all samples, although a slight increase is observed with decreasing deposition temperature, which may be attributed to the presence of Pb_Zr_ antisite defects at lower deposition temperatures (*53*–*55*).

The field-dependent evolution of the *P-E* hysteresis loops for PbZrO_3_ thin films at different deposited temperatures was summarized in fig. S4. The double-hysteresis loops of PZO (620) and PZO (580) thin films evolve from partial to complete AFE switching as the maximum applied field increases from 285 to 1000 kV/cm (fig. S4, A and B). The PZO (540) thin film exhibits FE-like hysteresis loops at low electric fields, with fluctuation appearing in the middle of the double-hysteresis loops as the electric fields increase and the overall polarization progressively enhanced (fig. S4C). This feature is more pronounced in PZO (500) thin film (Fig. 3E), which demonstrates the smooth FE hysteresis loop with nonzero remanent polarization of 1.8 and 3.8 μC/cm^2^ and saturation polarization of 15.9 and 20.2 μC/cm^2^ at low electric fields of 285 and 428 kV/cm, respectively. As the electric fields increase to 571, 714, and 857 kV/cm, the double-hysteresis loops resemble AFE behavior gradually appear at both ends of the single-hysteresis loops. Furthermore, under a large applied electric field of ±1000 kV/cm, the abnormal triple-hysteresis loop is exhibited, with a nonzero remanent polarization of 7.5 μC/cm^2^ and a saturation polarization of 52.2 μC/cm^2^. These results can be attributed to the presence of a ferrielectric phase with the triple dipole modulation configuration, as revealed by atomic-scale structural analysis. This emerging ferrielectric behavior contrasts with the intrinsic AFE ordering in pristine AFE PbZrO_3_ thin film, where the polarization occurs because of an electric field–induced phase transition from AFE (FE) to FE (AFE) when the field is applied (withdrawn). To validate the stability of FE switching under low electric fields, the butterfly-shaped *C-E* curve (Fig. 3F) exhibits two peaks at coercive fields, indicating that electric field–induced polarization switching can be achieved at low electric fields. Moreover, we used the switching spectroscopy piezoresponse force microscopy (SS-PFM) technique to detect the phase and amplitude signals of PZO (500) thin film under low electric fields. As shown in Fig. 3G, the single loop in the phase signal with a switching variation of ~180° at ±285 kV/cm demonstrates deterministic polarization reversal, while the butterfly-shaped profile in the amplitude signal confirms nonlinear electromechanical coupling. As the electric field increases, fig. S5A exhibits a triple-subloop feature of the phase signal at 1000 kV/cm, indicating that the phase has been reversed six times ~180°. The amplitude signal in fig. S5B also displays six distinct maxima, which reflect the variation of average piezoresponse strength during six phase reversals. Overall, the macroscopic *P-E/I-E/C-E* curves and local SS-PFM measurements demonstrate robust FE switching at low electric fields in the low-temperature–deposited PbZrO_3_ thin films, while additional field-driven transitions occur under large electric fields that produce the observed triple-hysteresis loop.

### Application validation of the negative ECE

**Fig. 4.**
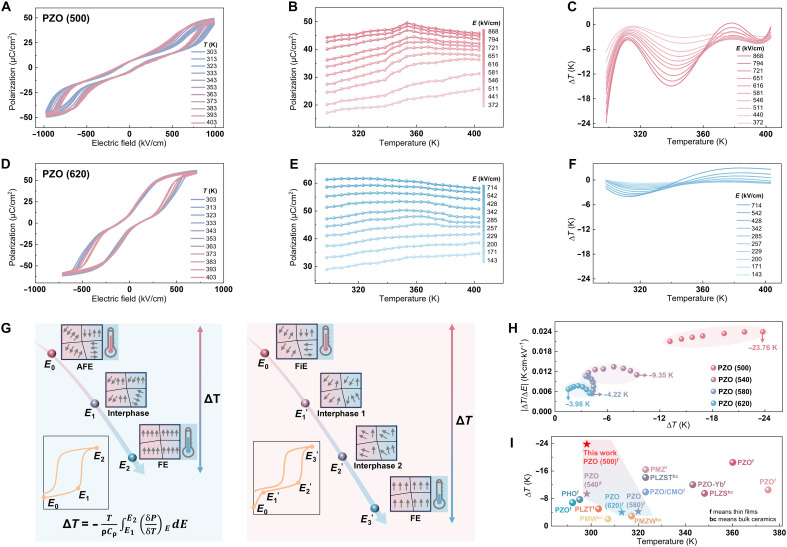
The ECE effect of PbZrO_3_ thin films. (**A**) The polarization-electric field (*P-E*) hysteresis loops of PZO (500) thin film with increased measure temperature. (**B**) The polarization-temperature (*P-T*) curves extracted from the positive branch of *P-E* loops under different electric fields in PZO (500) thin film. (**C**) The predicted temperature change (Δ*T*) as a function of temperature at selected applied fields in PZO (500) thin film. (**D**) The polarization-electric field (*P-E*) hysteresis loops of PZO (620) thin film with increased measure temperature. (**E**) The polarization-temperature (*P-T*) curves extracted from the positive branch of *P-E* loops under different electric fields in PZO (620) thin film. (**F**) The predicted temperature change (Δ*T*) as a function of temperature at selected applied fields in PZO (620) thin film. (**G**) The phase transition paths of PZO (620) and PZO (500) thin films during the positive electric field cycle. (**H**) The predicted Δ*T*_max_ and corresponding |Δ*T*/Δ*E*| values for PZO (500), PZO (540), PZO (580), and PZO (620) thin films under different electric fields. (**I**) The comparison of temperature change Δ*T* and corresponding temperatures in PZO-based thin films and bulk ceramics below 373 K. FiE, ferrielectric.

The triple-hysteresis PbZrO_3_ thin film enables multilevel phase transitions and enhances phase transition electric fields, offering the potential for superior energy storage performance, nonvolatile multistate memory properties, and the ECE. Here, we have focused on investigating the ECE, specifically evaluating the predicted temperature change Δ*T* in PbZrO_3_ thin films at different deposition temperatures using Maxwell’s relation (*56*)

$$\Delta T=-\frac{T}{\rho C_{\rho }}\int_{E_{1}}^{E_{2}}{\left(\frac{\delta P}{\delta E}\right)}_{E}\mathrm{dE}$$ (1)

where *T*, ρ, and *C*_ρ_ represent temperature, density, and heat capacity, respectively. We measured the temperature-dependent *P-E* loops of PZO (500) thin film under 868 kV/cm at an interval of 10 K from 303 to 403 K, presented in Fig. 4A. Figure S6A demonstrates that the saturation polarization initially increases and then decreases as temperature increases, while the intermediate hysteresis characteristics gradually diminish. Figure S6B displays the corresponding temperature-dependent *I-E* switching curves, wherein the switching current initially increases and then slightly decreases with increasing temperature, while the two current peaks near zero electric field gradually weaken. The evolution of the switching current aligns well with that of the *P-E* loops, suggesting that the polarization changes primarily originate from the displacement current. Figure 4B depicts the polarization-temperature (*P-T*) curves extracted from the positive branch of *P-E* loops under different electric fields. The polarization increases gradually with rising temperature under low electric fields, whereas the polarization initially increases and then decreases under high electric fields. Figure S6C shows the results of fitting a sixth-order polynomial to the *P-T* curves. The (δ*P*/δ*T*) values displayed in fig. S6D were calculated from the differential of the fitted *P-T* curves. The predicted temperature change Δ*T* and entropy change Δ*S* (Fig. 4C and fig. S6E) were obtained by integrating the (δ*P*/δ*T*) values and calculating according to formula (1) and (2) in the Supplementary Materials, respectively, where *E*_1_ was set to zero and *E*_2_ assumed 10 values between 372 and 868 kV/cm. The results indicate that the negative ECE predominates in the PZO (500) thin film, and the maximum predicted values at room temperature are Δ*T*_max_ ≈ −23.76 K and Δ*S*_max_ ≈ −26.3 J K^−1^ kg^−1^ under 868 kV/cm. The predicted negative ECE value of −23.76 K at room temperature highlights the promise of the triple-hysteresis design for solid-state cooling device applications. For comparison, the temperature-dependent *P-E* loops of PZO (620) thin film under 714 kV/cm at an interval of 10 K from 303 to 403 K, presented in Fig. 4D. Figure S7A shows that the saturation polarization tends to increase slightly at first and then decrease with increasing temperature. We used the identical data extraction and computational methods described above to derive the *P-T* curves and (δ*P*/δ*T*) values (Fig. 4E and fig. S7, B and C). Figure 4F and fig. S7D suggest that the negative ECE in the PZO (620) thin film reached the maximum value at 313 K under 714 kV/cm, with predicted Δ*T*_max_ ≈ −3.98 K and Δ*S*_max_ ≈ −4.2 J K^−1^ kg^−1^. These results demonstrate that triple-hysteresis PbZrO_3_ thin film exhibits greater cooling effect compared to double-hysteresis PbZrO_3_ thin film.

To elucidate the mechanism origin of enhanced negative ECE in the triple-hysteresis PbZrO_3_ thin film, Fig. 4E illustrates the phase transition paths of both during the positive electric field cycle. As shown in Fig. 4E (left), the initial state of double-hysteresis PbZrO_3_ thin film is the AFE state with quadruple (↑↑↓↓) periodicity antiparallel dipole arrangement. The applied electric field is increased to *E*_1_, corresponding to the interphase, where the dipoles gradually tilt and entropy increases and eventually transforms into the FE state as the electric field continues to rise. In this process, the combination of entropy change induced by dipole reversal and lattice structure alteration during AFE-to-FE phase transition collectively leads to the temperature change, denoted as Δ*T*. In contrast, as shown in Fig. 4E (right), an intermediate hysteresis embedded within the double-hysteresis loop results in the initial state of thin film being the ferrielectric state with the triple (↑↑↓) periodicity dipole arrangement. With increasing electric field to *E*_1_′ and *E*_2_′, this process undergoes two phase transitions corresponding to the interphase 1 and interphase 2, respectively, ultimately transforming into the FE phase upon reaching *E*_3_′. Distinct from conventional AFEs, two phase transitions occur during the applied electric field, which implies that the lattice structure experiences two substantial transformations. Moreover, according to Maxwell’s relations, the approach to enhance the negative ECE in AFEs lies in modulating the AFE-to-FE phase transition to increase the electric field required for this phase transformation. Based on the feature of the triple-hysteresis loop, our design strategy has enhanced the AFE-to-FE phase transition coercive electric field. Consequently, the combined effect of lattice structure alteration induced by two field-induced phase transitions and the increased AFE-to-FE phase transition electric field bring a large predicted temperature change Δ*T*.

For comparison, we used identical measurement and calculation methods to obtain the predicted temperature change Δ*T* and entropy change Δ*S* for PZO (540) and PZO (580), presented in figs. S8 and S9. In Fig. 4F, we extracted the predicted Δ*T*_max_ and calculated the corresponding |Δ*T*/Δ*E*| values for PZO (500), PZO (540), PZO (580), and PZO (620) thin films under different electric fields. The results indicate that the predicted Δ*T*_max_ enhanced from −3.98 to −23.76 K as the deposition temperature decreased, with the corresponding negative ECE strength from 0.006 to 0.024 K·cm·kV^−1^. It demonstrates that structural design strategy by reducing deposition temperature can effectively enhance the negative ECE in PbZrO_3_ thin films. Moreover, Fig. 4G compares the temperature change Δ*T* and corresponding temperatures of PZO-based thin films and bulk ceramics below 373 K (*57*–*68*). The results reveal that our triple-hysteresis PbZrO_3_ thin films exhibit exceptional negative ECE at room temperature, which holds substantial implications for practical cooling device applications.

## DISCUSSION

In summary, we devised a strategy of modulating the deposition temperature to obtain PbZrO_3_ thin films with progressive transformation from the double-hysteresis to the triple-hysteresis loop. The electrical properties of the latter demonstrate stable ferroelectricity at low electric fields and the ability to perform multilevel phase transitions with triple-hysteresis loop under large electric fields. Diffraction and photoelectron spectroscopy combined with electron microscopy characterizations attributed this behavior to the presence of Pb_Zr_ antisite defects acting as seeds for polar order, which induce the distinctive triple (↑↑↓) dipole modulation period configuration. To validate the functional potential of this behavior, we evaluated the ECE of triple-hysteresis PbZrO_3_ thin film based on Maxwell’s relations, which predicted temperature change Δ*T* reaching −23.76 K with an enhancement of ~600% over double-hysteresis PbZrO_3_ AFE thin films. This enhancement directly results from the improved phase transition electric field and the cumulative entropy gains associated with the multilevel phase transitions process. Our results establish a practical design paradigm for embedding stable FE switching within AFE matrices and open avenues for developing high-density energy storage, nonvolatile multistate memory, and highly efficient switching devices.

## MATERIALS AND METHODS

### Sample preparation

The high-quality PbZrO_3_/SrRuO_3_ (PZO/SRO) heterostructures were synthesized on (001)-oriented SrTiO_3_ (STO) single-crystal substrates via PLD [RP-HT-102, purchased from Arrayed Materials (China) Co. Ltd.]. The SrRuO_3_ bottom electrode layer was first deposited on the SrTiO_3_ (001) substrate at 680°C with a dynamic oxygen pressure of 80 mtorr. Subsequently, PbZrO_3_ was deposited at temperatures of 620°, 580°, 540°, and 500°C with a dynamic oxygen pressure of 100 mtorr, respectively. After deposition, the thin films were cooled down to room temperature at a rate of 10°C min^−1^ in an oxygen pressure of 100 mtorr. Note that the laser energy and repetition rate were set as 350 mJ and 10 Hz during SrRuO_3_ and PbZrO_3_ thin film deposition. Last, Au electrodes with diameters of 100 μm were deposited by sputtering through a shadow mask.

### Crystal structure characterizations

The symmetric XRD 2θ-ω scans of the heterostructures were performed with an x-ray diffractometer (SmartLab 9 kW, Rigaku, Tokyo, Japan) equipped with Cu-Kα radiation. 2θ-ω scan was conducted along the out-of-plane direction to examine the purity of epitaxial thin films at a speed of 2° min^−1^. The chemical valence states of the PbZrO_3_ thin films were characterized via XPS (Escalab Xi^+^).

### Transmission electron microscopy

The TEM samples used for in situ TEM were prepared using a focus ion beam scanning electron microscopy (Helios G4 UX, Thermo Fisher Scientific). Cross-sectional lamellas lifted out using focused ion beam (FIB) were thinned down to ~200-nm thickness at an accelerating voltage of 30 kV with a decreasing current from the maximum 2.5 nA, followed by fine polishing at 5- and 2-kV milling down to ~50 nm, and 1-kV setting was used for the final milling. The atomic structures of the films were characterized using a JEM ARM300 (JEOL, Tokyo, Japan) transmission electron microscope operated at 300 kV and equipped with double spherical aberration (Cs) correctors and EDS. The collection angle for HAADF-STEM imaging was 90 to 370 mrads. The atomic-resolution HAADF-STEM images were quantitatively analyzed using CalAtom software to extract precise atomic column intensities and positions.

### Electrical properties measurements

The electrical measurements (*P-E/I-E/C-E*) were performed with a TF3000 analyzer (aixACCT) on a Semishare high-precision probe station (Semishare E4). The *P-E/I-E/C-E* loops were characterized under 5-kHz dc electric field. The SS-PFM mode was used with pulsed triangular dc driving voltage (*V*_dc_), where the dc pulse width was set to 10 to 15 ms and the rise time of each pulse was fixed at 0.5 ms. To avoid the alteration of polarization states by the ac detection voltage, we used the detection voltages in the range from 0.2 to 0.4 *V*_ac_ to detect the piezoresponse signal during and after each dc pulse to ensure the reliability of the measured data. To void the ambiguity from the electrostatic force for on-field (dc voltage is on) measurements, we focused on the off-field (remanent) data for our analyses.
