# Supplementary material for: Antiferroelectric thin films embedded with ferroelectric switching loop for giant negative electrocaloric effect
Source: Sci Adv. 2026 Jul 1;12(27):eaed5447. doi: 10.1126/sciadv.aed5447 (PMC13322268; doi:10.1126/sciadv.aed5447)
Supplement: Supplementary file 1 — Figs. S1 to S9 [file sciadv.aed5447_sm.pdf]

Science Advances
AAAS

Supplementary Materials for

Antiferroelectric thin films embedded with ferroelectric switching loop for giant negative electrocaloric effect

Peipei Su *et al.*

Corresponding author: Gaokuo Zhong, gkzhong@hnu.edu.cn; Xiangli Zhong, xlzhong@xtu.edu.cn; Ke Qu, kqu@chem.ecnu.edu.cn

Sci. Adv. **12**, eaed5447 (2026)
DOI: 10.1126/sciadv.aed5447

This PDF file includes:

Figs. S1 to S9

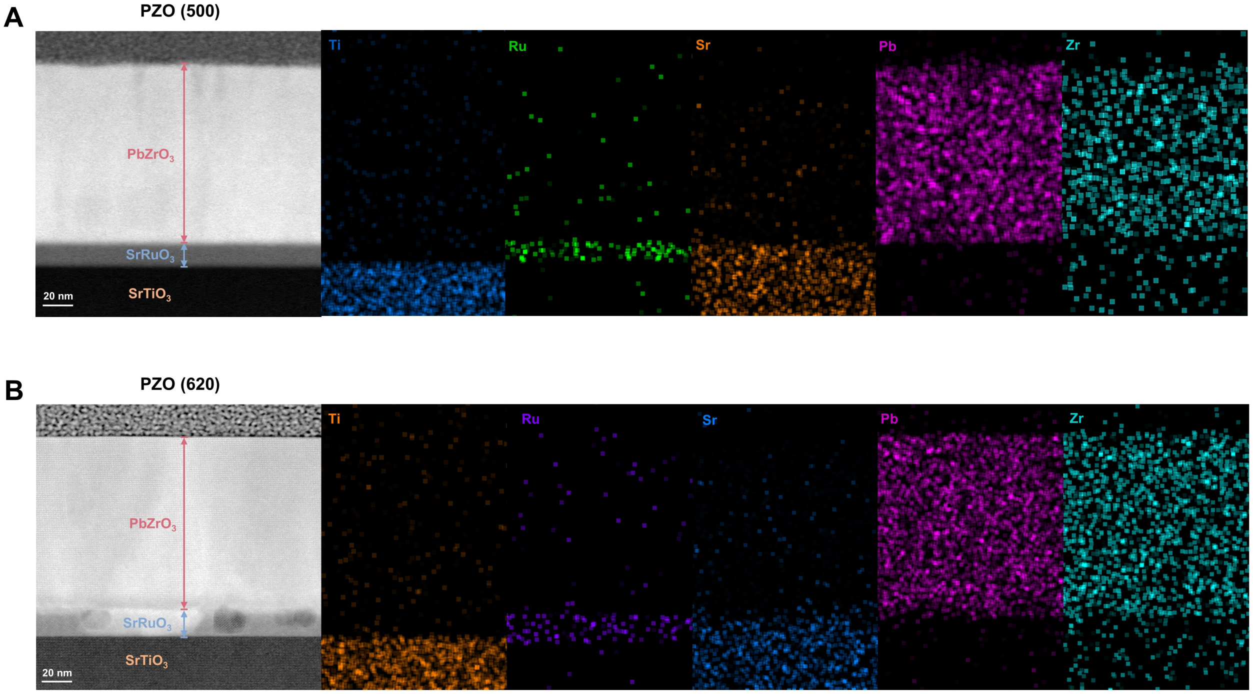

Fig. S1. STEM images and EDS elemental mappings. (**A** and **B**) Large-scale cross-sectional dark-field STEM images with the corresponding EDS element mappings of the PZO (500) and PZO (620) thin films, respectively. (The left panel of fig. S1A is identical to the left panel of Fig. 1G)

To further recognize the microstructures of $PbZrO_3$ thin films deposited at different temperatures, fig. S1 presents the cross-sectional dark-field transmission electron microscopy (TEM) images of PZO (500) and PZO (620) thin films alongside corresponding elemental energy-dispersive spectroscopy (EDS) analysis. Large-area TEM images with no visible defects indicate that both samples exhibit high epitaxial quality and sharp heterointerfaces. Meanwhile, the EDS analysis of Ti, Ru, Sr, Pb and Zr elemental spectra confirm chemical uniformity.

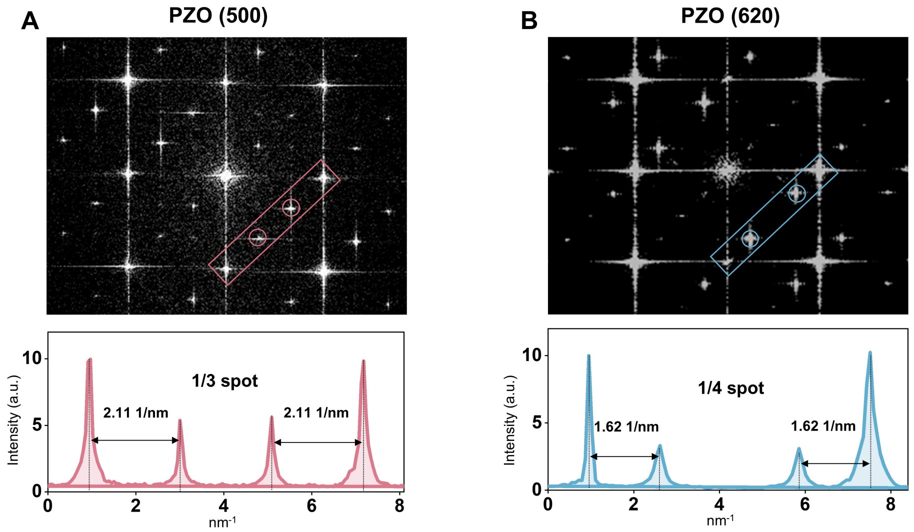

Fig. S2. The fast Fourier transform (FFT) modes for two thin films. (**A** and **B**) FFT patterns and corresponding intensity profile of the selected rectangular regions for PZO (500) and PZO (620) thin films, respectively.

The upper of fig. S2 corresponds to the fast Fourier transform (FFT) patterns of HAADF-STEM images for PZO (500) and PZO (620) thin films respectively, both revealing the additional 1/x{011} diffraction spots. The bottom of fig. S2 details the positions and intensity distributions of the main and additional diffraction spots. The specific distance calculation indicate that the distance between the main and additional diffraction spots in the PZO (500) and PZO (620) thin films were 2.11 and 1.62 nm^{-1}, respectively. Based on these distances, the additional diffraction spots are estimated to be located at approximately the 1/3 (2/3) and 1/4 (3/4) positions, and the intensity of additional diffraction spots in the former is greater.

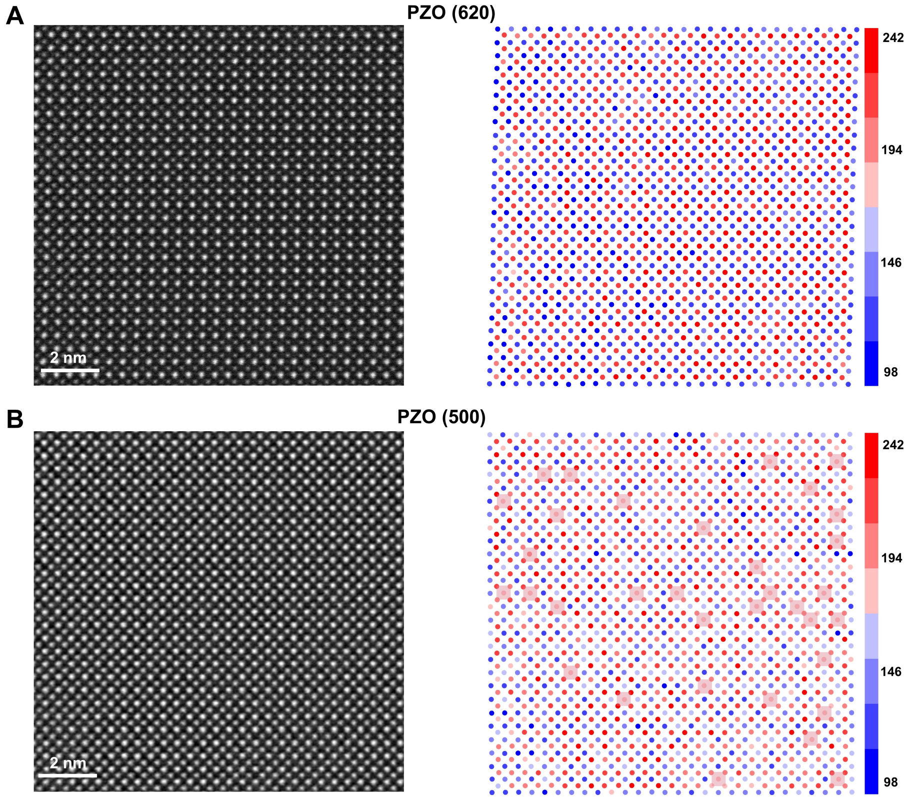

Fig. S3. The HAADF-STEM images and intensity of atomic columns. (**A** and **B**) Atomic-scale HAADF-STEM images and corresponding Pb and Zr atomic intensity statistics of PZO (620) and PZO (500) thin films, respectively.

To gain deeper insight into the ferrielectric phase, we statistically analyzed the atomic intensity of large region HAADF-STEM images for PZO (620) and PZO (500) thin films. Figure S3 displays the corresponding atomic intensity maps for both HAADF-STEM images. Since the atomic intensity in HAADF images depends on the atomic number (Z), a color map is used, with red representing atoms with a higher Z, e.g., Pb atoms at A-sites, and blue representing atoms with a lower Z, e.g., Zr atoms at B-sites. It should be noted that abnormally high intensities (marked by light red boxes) appear on certain atomic columns at the B-sites of the PZO (500) thin film, which are normally occupied by lighter Zr atoms. This indicates the presence of heavier Pb ions at several Zr-like sites.

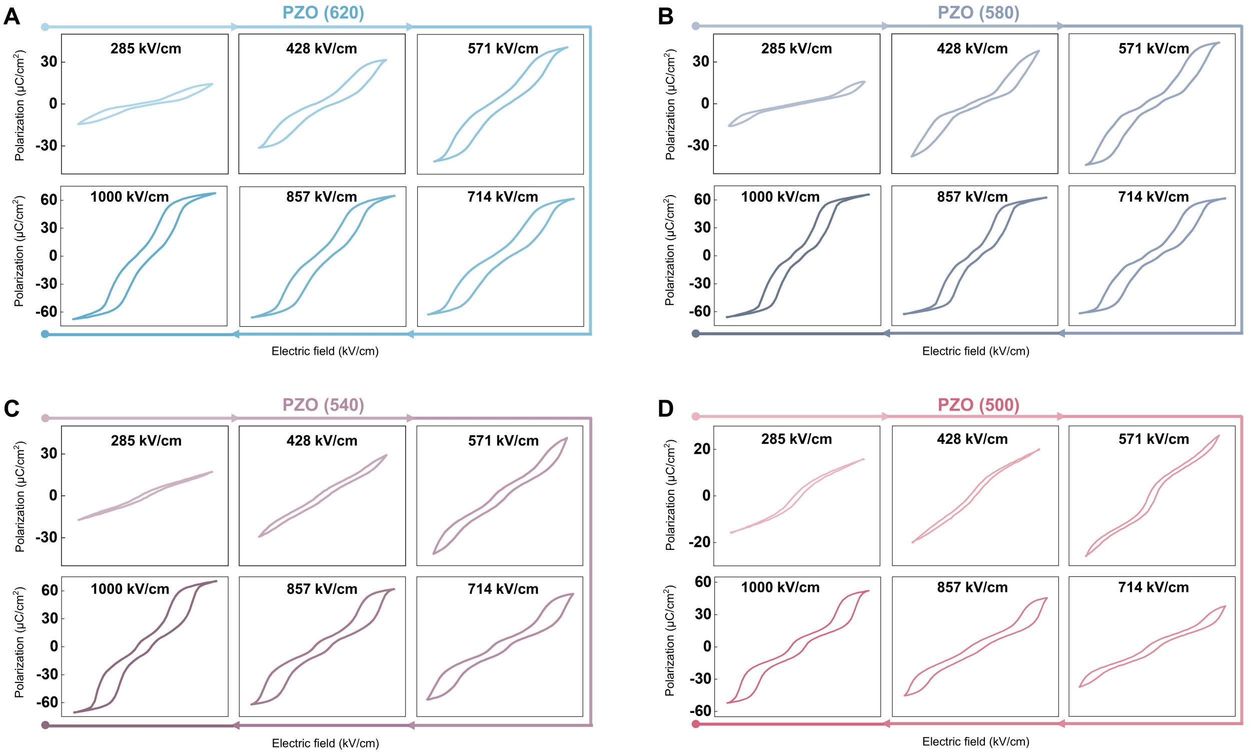

Fig. S4. The polarization-electric field (*P-E*) hysteresis loops in $PbZrO_3$ thin films. (**A** to **D**) Evolution of polarization-electric field (*P-E*) hysteresis loops with electric fields increase in the PZO (620), PZO (580), PZO (540), and PZO (500) thin films, respectively.

The double-hysteresis loops of $PbZrO_3$ thin films deposited at 620 °C and 580 °C (fig. S4, A and B), gradually transition from incomplete to complete polarization as the electric fields increase from 285 kV/cm to 1000 kV/cm. The PZO (540) thin film exhibits ferroelectric-like hysteresis loops at low electric fields (fig. S4C), with fluctuation appearing in the middle of the double-hysteresis loops as the electric fields increase, while the polarization value progressively enhanced. This feature is more significant in PZO (500) thin film, fig. S4D demonstrating the smooth ferroelectric hysteresis loop with nonzero remanent polarization of 1.8, 3.8 μC/cm^2 and saturation polarization of 15.9, 20.2 μC/cm^2 at low electric fields of 285 and 428 kV/cm, respectively. As the electric fields increase to 571, 714 and 857 kV/cm, the double-hysteresis loops resemble antiferroelectric behavior gradually appear at both ends of the single-hysteresis loops. Furthermore, under a large applied electric field of ±1000 kV/cm, the abnormal triple-hysteresis loop is exhibited, with a nonzero remanent polarization of 7.5 μC/cm^2 and a saturation polarization of 52.2 μC/cm^2.

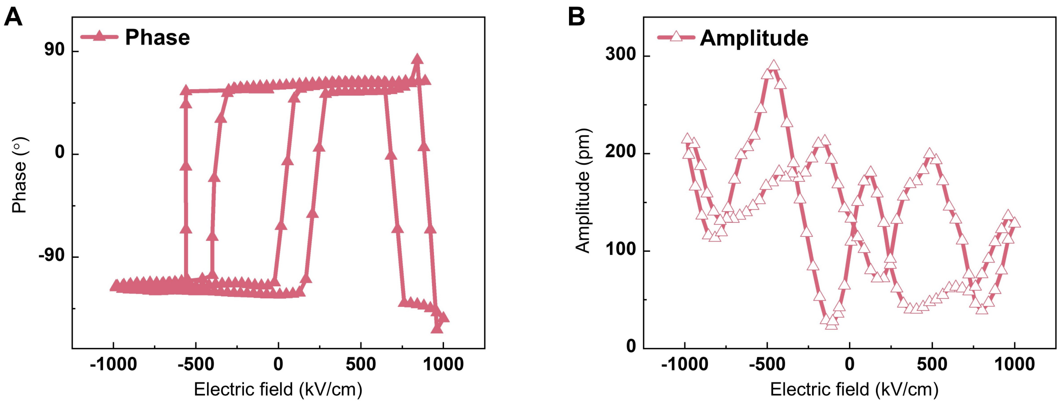

Fig. S5. Electrical properties of the PZO (500) thin films under large electric field. (**A** and **B**) The phase and amplitude hysteresis loops of the PZO (500) thin film at 1000 kV/cm.

To investigate the microscopic domain switching dynamics of the PZO (500) thin film under large electric fields, we employed SS-PFM to probe the phase and amplitude responses. As shown in fig. S5A, the phase signal of the PZO (500) thin film exhibits a triple-subloop feature at 1000 kV/cm, indicating that the phase has been reversed six times approximately 180°. The amplitude signal in fig. S5B also displays six distinct maxima, which reflect the variation of average piezoresponse strength during six phase reversals. These results further validate the triple-hysteresis loop along with the six-peak characteristics observed in the *I-E* and *C-E* curves, revealing that PZO (500) thin film can achieve stable multi-step polarization switching under large electric fields.

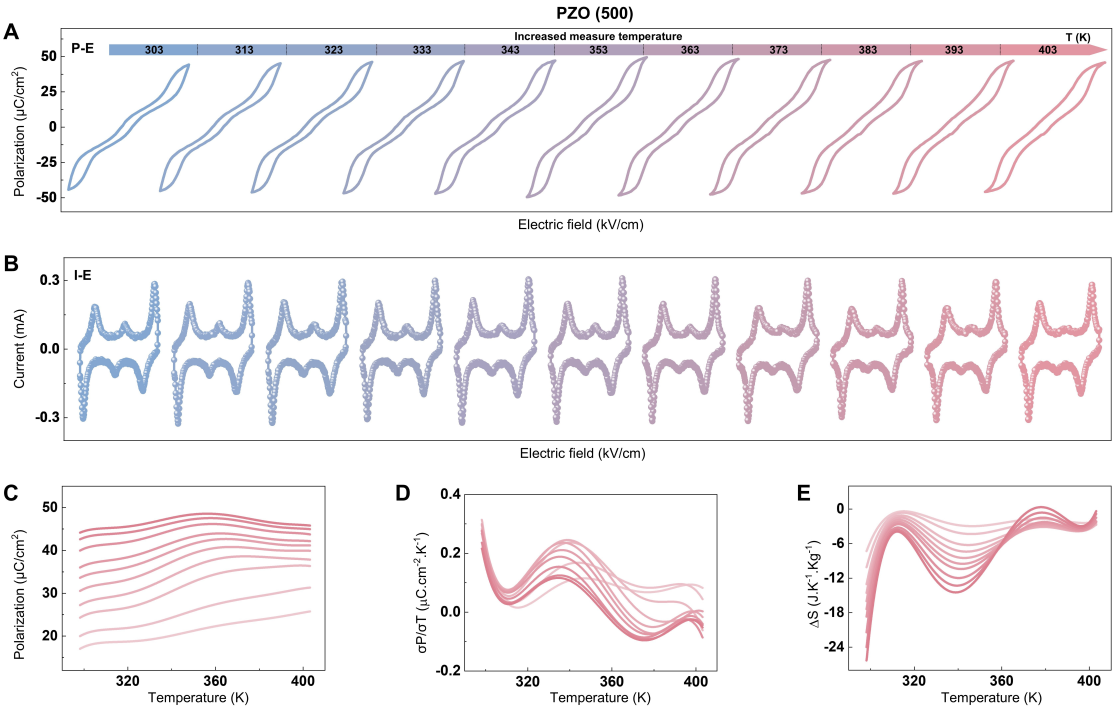

Fig. S6. The ECE effect of PZO (500) thin film. (**A**) The polarization-electric field (*P-E*) hysteresis loops of PZO (500) thin film with increased measure temperature. (**B**) The corresponding current-electric field (*I-E*) switching curves of PZO (500) thin film with increased measure temperature. (**C**) The sixth-order polynomial fit of the *P-T* curves. (**D**) The values of (δP/δT) calculated from the differential of the fitted *P-T* curves. (**E**) The predicted entropy change (ΔS) as a function of temperature at selected applied fields in PZO (500) thin film.

Here, we calculated the predicted temperature change ΔT and entropy change ΔS in $PbZrO_3$ thin films at different deposition temperatures using Maxwell's relations:

$$\Delta T = -\frac{T}{\rho C_\rho}\int_{E_1}^{E_2}\left(\frac{\delta P}{\delta T}\right)_E dE \tag{1}$$

$$\Delta S = -\frac{1}{\rho}\int_{E_1}^{E_2}\left(\frac{\delta P}{\delta T}\right)_E dE \tag{2}$$

where T, ρ, C_ρ, E_1 and E_2 represent the temperature, the density, the heat capacity, the initial and final applied electric fields, respectively. This work adopts the values reported in the literature for density ρ of approximately 8.22 g/cm^3 and heat capacity C_ρ of around 330 J/K/kg. We measured the temperature-dependent *P-E* loops of PZO (500) thin film under 868 kV/cm at an interval of 10 K from 303 K to 403 K, and the results for every 10 K were presented in fig. S6A. Figure S6B displays the corresponding temperature-dependent *I-E* switching curves, wherein the switching current initially increases and then slightly decreases with increasing temperature, while the two current peaks near zero electric field gradually weaken. Fig. S6C shows the results of fitting a sixth-order polynomial to the *P-T* curves in Fig. 4B. The (δP/δT) values (fig. S6D) were calculated from the differential of the fitted *P-T* curves shown in fig. S6C. The predicted temperature change ΔT and entropy change ΔS (Fig. 4C and fig. S6E) were obtained by integrating the (δP/δT) values and calculating according to formula (1) and (2), respectively, where E_1 was set to zero and E_2 assumed ten values between 372 kV/cm and 868 kV/cm.

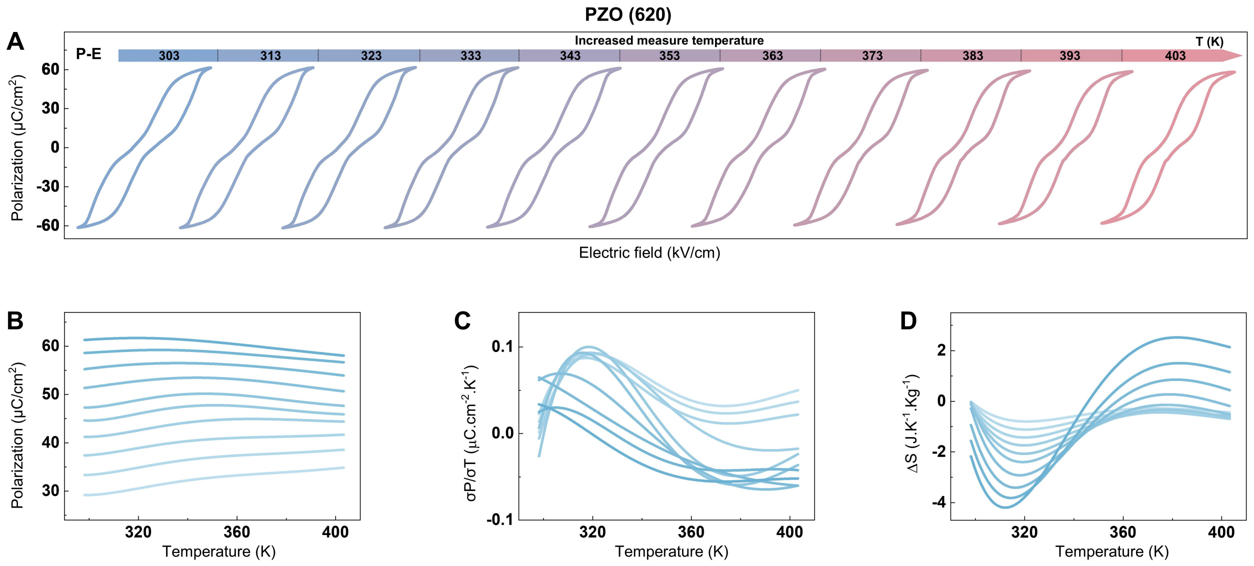

Fig. S7. The ECE effect of PZO (620) thin film. (**A**) The polarization-electric field (*P-E*) hysteresis loops of PZO (620) thin film with increased measure temperature. (**B**) The sixth-order polynomial fit of the *P-T* curves. (**C**) The values of (δP/δT) calculated from the differential of the fitted *P-T* curves. (**D**) The predicted entropy change (ΔS) as a function of temperature at selected applied fields in PZO (620) thin film.

The predicted temperature change ΔT and entropy change ΔS of the PZO (620) thin film were calculated employing the same calculation method as that used for the above mentioned of PZO (500) thin film.

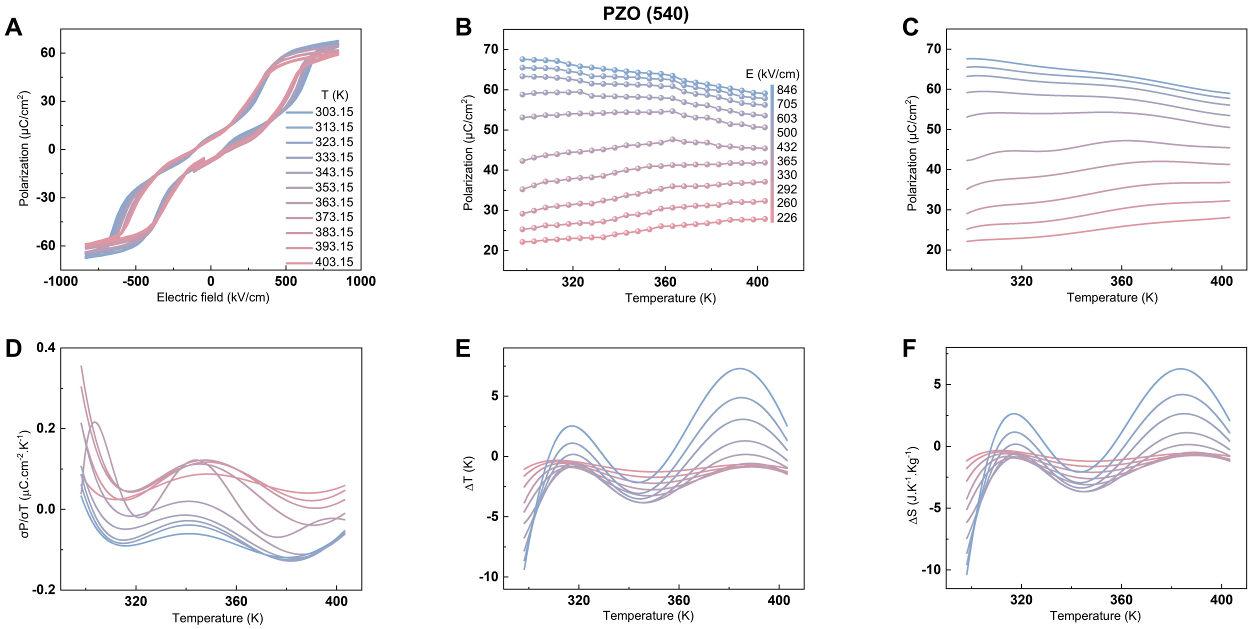

Fig. S8. The ECE effect of PZO (540) thin film. (**A**) The polarization-electric field (*P-E*) hysteresis loops of PZO (540) thin film with increased measure temperature. (**B**) The polarization-temperature (*P-T*) curves extracted from the positive branch of *P-E* loops under different electric fields. (**C**) The sixth-order polynomial fit of the *P-T* curves in (B). (**D**) The values of (δP/δT) calculated from the differential of the fitted *P-T* curves in (C). (**E** and **F**) The predicted temperature change (ΔT) and entropy change (ΔS) as a function of temperature at selected applied fields in PZO (540) thin film.

The predicted temperature change ΔT and entropy change ΔS of the PZO (540) thin film were calculated employing the same calculation method as that used for the above mentioned of PZO (500) thin film.

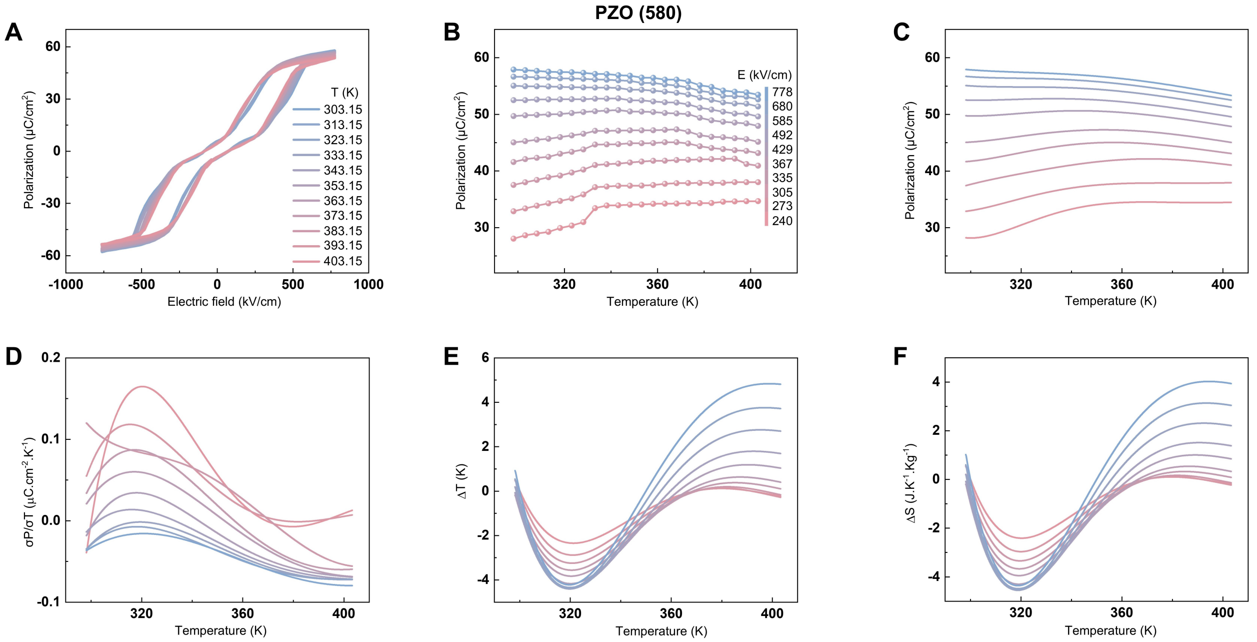

Fig. S9. The ECE effect of PZO (580) thin film. (**A**) The polarization-electric field (*P-E*) hysteresis loops of PZO (580) thin film with increased measure temperature. (**B**) The polarization-temperature (*P-T*) curves extracted from the positive branch of *P-E* loops under different electric fields. (**C**) The sixth-order polynomial fit of the *P-T* curves in (B). (**D**) The values of (δP/δT) calculated from the differential of the fitted *P-T* curves in (C). (**E** and **F**) The predicted temperature change (ΔT) and entropy change (ΔS) as a function of temperature at selected applied fields in PZO (580) thin film.

The predicted temperature change ΔT and entropy change ΔS of the PZO (580) thin film were calculated employing the same calculation method as that used for the above mentioned of PZO (500) thin film.
